# Obsessive Compulsive Disorder Networks: Positron Emission Tomography and Neuropsychology Provide New Insights

**DOI:** 10.1371/journal.pone.0053241

**Published:** 2013-01-11

**Authors:** Bruno Millet, Thibaut Dondaine, Jean-Michel Reymann, Aurélie Bourguignon, Florian Naudet, Nematollah Jaafari, Dominique Drapier, Valérie Turmel, Habiba Mesbah, Marc Vérin, Florence Le Jeune

**Affiliations:** 1 University Department of Adult Psychiatry, Guillaume Régnier Hospital, Rennes, France; 2 EA 4712 “Behaviour and Basal Ganglia” Research Unit, Universitary Hospital and University of Rennes 1, Rennes, France; 3 Clinical Investigation Centre, INSERM 0203, Clinical Pharmacology Unit, Universitary Hospital and University of Rennes 1, Rennes, France; 4 INSERM, Experimental and Clinical Neurosciences Laboratory, Team Psychobiology of Compulsive Disorders, Poitiers, France; 5 CIC INSERM U 802, Universitary Hospital of Poitiers and University of Poitiers, Poitiers, France; 6 Unité de recherche clinique intersectorielle en psychiatrie du Centre Hospitalier Henri Laborit, Poitiers, France; 7 Department of Medical Information, Eugène Marquis Centre, rue de la Bataille Flandres Dunkerque, Rennes, France; 8 Neurology Department, Pontchaillou Hospital, Rennes University Hospital, rue Henri Le Guilloux, Rennes, France; 9 Nuclear Medicine Department, Eugène Marquis Centre, rue de la Bataille Flandres Dunkerque, Rennes, France; Bellvitge Biomedical Research Institute-IDIBELL, Spain

## Abstract

**Background:**

Deep brain stimulation has shed new light on the central role of the prefrontal cortex (PFC) in obsessive compulsive disorder (OCD). We explored this structure from a functional perspective, synchronizing neuroimaging and cognitive measures.

**Methods and Findings:**

This case-control cross-sectional study compared 15 OCD patients without comorbidities and not currently on serotonin reuptake inhibitors or cognitive behavioural therapy with 15 healthy controls (matched for age, sex and education level) on resting-state ^18^FDG-PET scans and a neuropsychological battery assessing executive functions. We looked for correlations between metabolic modifications and impaired neuropsychological scores. Modifications in glucose metabolism were found in frontal regions (orbitofrontal cortex and dorsolateral cortices), the cingulate gyrus, insula and parietal gyrus. Neuropsychological differences between patients and controls, which were subtle, were correlated with the metabolism of the prefrontal, parietal, and temporal cortices.

**Conclusion:**

As expected, we confirmed previous reports of a PFC dysfunction in OCD patients, and established a correlation with cognitive deficits. Other regions outside the prefrontal cortex, including the dorsoparietal cortex and the insula, also appeared to be implicated in the pathophysiology of OCD, providing fresh insights on the complexity of OCD syndromes.

## Introduction

Deep brain stimulation (DBS), which has proved to be an effective treatment for obsessive-compulsive disorder (OCD) [1], has shed new light on this pathology and its underlying mechanisms. In particular, ^18^FDG-PET has revealed metabolic modifications in the wake of implantation [2], prompting us to take a fresh look at resting glucose metabolism in patients suffering from OCD without comorbidities.

OCD is a severe and debilitating neuropsychiatric disorder. Over the past 20 years, several findings have suggested that it could be due to a dysfunction of the cortico-striatal-thalamo-cortical (CSTC) circuitry [3]. Functional neuroimaging studies have largely supported this hypothesis. Baxter et al. [4] were the first to compare a group of patients suffering from OCD with a sample of healthy participants. They demonstrated bilateral hyperactivity of the orbitofrontal cortex (OFC), in addition to predominantly right-sided hyperactivity of the caudate nucleus. Since then, many ^18^FDG-PET studies, as well as a number of provocation studies using H_2_^15^O2 or functional MRI, have replicated these findings, reporting either left-sided, right-sided, or bilateral prefrontal cortex (PFC) hypermetabolism in OCD [4], [5], [6], [7].

Baxter's model postulates an imbalance between an overactive direct striatopallidal pathway in charge of the execution of routines and an indirect striatopallidal circuit. Schwartz's model [8], on the other hand, postulates a major filtering role for the head of the ventral caudate, in charge of selecting and generating new patterns of activity in response to significant relevant behavior. In his model, a dysfunctional ventral caudate controls the activity of two cortical regions, the OFC and the anterior cingulate cortex (ACC), both structures known to play a role in detecting emotional information.

A previous study of resistant OCD by our team [2] showed that a decrease in scores on the Yale-Brown Obsessive Compulsive Scale (Y-BOCS) was correlated with a decrease in PFC metabolism during subthalamic nucleus (STN) stimulation. This result strongly suggests that the efficacy of this technique is related to the reduced metabolism of the PFC and, more especially, of the OFC, which may therefore be a promising treatment target. Based on this finding, we hypothesized that OCD is related to a dysfunction not just in the basal ganglia, but also in the PFC. This practical consideration focused our attention on the PFC and thus on the executive functions that it is known to mediate. Several of these functions have been found to be impaired in OCD, in line with the OFC hypermetabolism, including decision-making [9] and reversal leaning [10].

The aim of this study was thus to explore executive functions in severe OCD patients without any obvious comorbidities in relation to whole-brain glucose metabolism at rest.

## Materials and Methods

### Study population

The protocol was approved by an institutional review board and written informed consent was obtained from each participant after a complete description of the study.

Fifteen outpatients with severe OCD and 15 healthy controls (HC) took part in this case-control cross-sectional study. Patients were recruited from the University Department of Adult Psychiatry in Rennes (France). They were deemed to be eligible for inclusion if they were adults and had received a primary diagnosis of OCD in accordance with the criteria of the Diagnostic and Statistical Manual of Mental Disorders, fourth edition (text revised) (DSM-IV TR) [11] and established with the use of the Diagnostic Interview for Genetic Studies [12]. They also had to have a disease duration of more than 5 years, and scores above 20 on the Y-BOCS [13], below 40 on the Global Assessment of Functioning (GAF) scale [11], and above 4 for severity of illness on the Clinical Global Impression (CGI) scale [14].

Additional inclusion criteria were right-handedness, and being without serotonin reuptake inhibitors for at least one month prior to the study and without cognitive-behavioural therapy for the past year.

Key exclusion criteria were the presence of significant medical illness, cognitive impairment (Mattis <130 [15]), suicidal ideation, as assessed by Item 10 (score ≥2) of the Montgomery Asberg Depression Rating Scale (MADRS) [16], moderate to severe depressive symptoms (MADRS score >20), DSM-IV Axis I (apart from general anxiety disorders) and Axis II disorders [17], a score above 14 on the Brown Assessment of Beliefs Scale (BABS) [18] to rule out any delusional component of the obsessive belief, and pregnancy for women.

The HC group consisted of 15 right-handed participants recruited from the hospital and the University of Rennes, matched for age, sex and education level. Pregnancy was an exclusion criterion for women. These HC had no history of neurological disease, head injury or alcohol abuse. A structural clinical interview for nonpatients [19] allowed us to exclude participants with any past or present psychiatric disorders.

Using the Y-BOCS checklist, OCD patients were divided into checkers, washers and hoarders, according to their predominant symptoms.

### Neuropsychological assessment

Patients and healthy controls underwent the same neuropsychological tests to assess their cognitive abilities:

#### Verbal memory (Hopkins test [20])

In each trial, a list of 12 words belonging to one of three semantic categories (4 words per category) was read out three times to the participants, who then had to perform an immediate free recall task. Twenty minutes later, participants performed a delayed free recall task, which was immediately followed by a recognition memory task. In this task, participants listened to a list of 24 words, with 12 words taken from the test list and 12 distracters. They were asked to recognize the 12 words belonging to the test list.

#### Visuospatial memory (Rey–Osterrieth Complex Figure Test, RCFT [21], [22])

This test assessing visuospatial constructional ability and visual memory consisted of a copy trial (max. score 36), followed by two recall trials 3 and 30 min later (max. score 36 for each trial).

#### Verbal fluency [23]

Two tasks were administered to participants. In the semantic task, participants were asked to say as many words belonging to the *animal* semantic category as possible in the space of 2 min. In the phonemic task, participants were asked to say as many words beginning with the letter *P* as possible in the space of 2 min. Performance was assessed by the number of different words produced within the allotted time.

#### Flexibility (Trail Making Test [24])

Participants had to join up 25 numbered circles randomly arranged on a page in ascending order (Part A) and 25 numbered and lettered circles in alternating order (Part B). Performance was assessed by the time (in seconds) it took them to complete each part (A and B). We also counted the number of errors for each part.

#### Response switching (Object Alternation Task [25])

In a computerized task, participants had to detect a coin hidden under one of two boxes (black vs. white). Following a correct response, the coin was put under the other box, whereas it remained under the same box if the previous response was incorrect. We counted the number of trials needed to solve the flexibility problem, the number of errors and the number of correct answers.

#### Inhibition (Stroop test [26])

A 100-item version was administered to participants. In the first phase (colour condition, C), participants were instructed to name the colour of each column of dots as quickly as possible without making any mistakes. In the second phase (reading condition, R), participants were instructed to read the name of colour of each column of dots as quickly as possible without making any mistakes. In the third phase (interference condition, CR), participants were shown a sheet of paper with 100 colour names printed in a colour different from the word itself. They were instructed to name the colour of the ink of each word as quickly as possible without making any mistakes. Three colour names were used to construct the list (*red*, *blue*, *green*).

Performances were assessed by the number of correct answers provided within 45 s for each phase, and an interference index calculated as follows: I = CR - ((C×R)/(C+R)).

#### Planning (Tower of London [27])

This test consisted of two boards with pegs, and beads in three different colours (red, green and blue). The examiner used the beads and the boards to present the participants with problem-solving tasks. Participants had to resolve a series of 20 problems. We counted the number of correctly solved trials, the time taken for the first shot and the total completion time.

### PET imaging procedure

In the same week as the neuropsychological assessment, all participants underwent an ^18^F-FDG-PET scan in a resting state. PET measurements were performed using a dedicated Discovery ST PET scanner (GEMS, Milwaukee, USA) in 2D mode with an axial field of view (FOV) of 15.2 cm and axial resolution of 3.91 mm. A 222–296 MBq injection of ^18^F-FDG was administered intravenously under standardized conditions (in a quiet, dimly lit room with the patient's eyes and ears open). During the acquisition, the participant's head was immobilized using a head-holder. A cross-laser system was used to ensure stable and reproducible positioning. A 20-min 2D emission scan was performed 30 minutes post injection. Attenuation correction was provided by a CT scan prior to the emission scan. These studies were performed with the participants positioned at the centre of the FOV. Following corrections for scatter, dead time and random, PET images were reconstructed by 2D filtered back-projection providing 47 contiguous transaxial 3.75 mm thick slices.

### Analysis of neuropsychological data

Neuropsychological data were analyzed with SAS 9.1 software (SAS Institute, Cary, NC, USA). OCD patients and HC were compared using the Wilcoxon signed-rank test for continuous variables, and McNemar's test for categorical variables. For continuous variables, we report the means (± *SD*), and for categorical variables, we supply the number of patients in each category (and the corresponding percentage).

### Analysis of PET data

PET data were analyzed using SPM2 software (Wellcome Dept of Cognitive Neurology, London, UK) implemented in MATLAB, Version 7 (Mathworks Inc., Sherborn, MA, USA). Statistical parametric maps are spatially extended statistical processes that are used to characterize specific regional effects in imaging data. SPM combines the general linear model (to create the statistical map) with Gaussian field theory in order to draw statistical inferences about regional effects [28]. All the participants' images were first realigned and spatially normalized into standard stereotactic space according to MNI space. An affine transformation was performed to determine the 12 optimum parameters for registering the brain images onto the template, and the subtle differences between the transformed image and the template were then removed using a nonlinear registration method. Finally, spatially normalized images were smoothed using an isotropic 12-mm full width at half-maximum Gaussian kernel to compensate for interindividual anatomical variability and render the imaging data more normally distributed.

### Cerebral metabolic differences between OCD patients and HC

The effects of overall metabolism were removed by normalizing the count of each voxel to the total brain count (proportional scaling in SPM). Significant changes in regional cerebral metabolism in the 15 OCD patients were then estimated by comparing their PET images with those of the 15 HC using the “compare-populations one scan/subject” routine, which performed a fixed-effects simple *t* test for each voxel. The effect of overall differences in blood flow was removed using proportional scaling, with the global mean set at 50 and threshold masking set at 0.8. Clusters of a minimum of 50 contiguous voxels, with a threshold *p*<0.001 (two-tailed uncorrected) were considered to be significantly different.

All coordinates reported here are based on the Talairach atlas and were transformed by applying procedures developed by Matthew Brett (http://www.mrc-cbu.cam.ac.uk/Imaging).

### Metabolic-neuropsychological correlation analyses

We only looked for correlations between cerebral glucose metabolism and executive functions that had been found to be impaired in the OCD patients compared with the HC. Using SPM, we tested a general linear “single subject, covariates only” model for every voxel to identify those regions that correlated significantly with neuropsychological scores. These correlations (*p* = 0.001, uncorrected, cluster size: 50) were only calculated for OCD patients: first, between decreased cerebral glucose metabolism and the neuropsychological data, second, between increased cerebral glucose metabolism and the neuropsychological data.

## Results

### Initial characteristics and neuropsychological data

The OCD patient group consisted of 10 checkers, three washers and two hoarders. The initial characteristics and neuropsychological performances of OCD patients compared with HC are set out in **Table 1**. The patients' verbal fluency score (animal names) was significantly lower than that of HC. In the Stroop test, OCD patients gave fewer correct responses when naming the colour and when naming the incongruous colour of the printed word. Their interference score was also lower than that of HC. OCD patients took significantly longer to complete the Tower of London task. No significant differences were observed in the other tests.

**Table 1:** Initial characteristics and neuropsychological performances of OCD patients and healthy controls.

Variable	OCD patients (*n* = 15)	Healthy controls (*n* = 15)	*p*-value
***Demographic characteristics***			
Sex ratio (female/male)	5/10	5/10	NA
Age (years)	36+/−13	37+/−13	NA
Education (years)	13+/−2	12+/−2	NA
***Clinical characteristics***			
Y-BOCS	27.4+/−5.7	NA	NA
GAF	33+/−3	NA	NA
CGI	5+/−1	NA	NA
Mattis	138+/−4	140+/−2	0.142
MADRS	12+/−8	1+/−2	**<0.001**
***Neuropsychological performance***			
Hopkins Test total (score)	24.8+/−5.2	27.7+/−4.4	0.070
Hopkins Test delayed recall (score)	9.3+/−2.1	9.4+/−2.5	0.838
Hopkins Test recognition (score)	11.3+/−1.0	11.3+/−1.3	0.873
Word fluency v (n)	14.4+/−4.3	17.7+/−4.7	0.052
Word fluency p (n)	21.9+/−6.0	22.9+/−7.0	0.718
Word fluency animals (n)	31.1+/−7.5	38.9+/−7.7	**0.009**
RCFT copy (score)	35.2+/−1.1	35.7+/−0.7	0.089
RCFT immediate recall (score)	20.4+/−9.2	23.1+/−8.0	0.441
RCFT recall delayed (score)	20.0+/−9.3	22.1+/−7.2	0.534
TMT A correct answers (n)	25+/−0	25+/−0	NA
TMT A time (s)	31.7+/−15.4	25.7+/−8.4	0.164
TMT B correct answers (n)	24.5+/−1.1	24.5+/−1.4	1.000
TMT B time (sec)	89.4+/−55.6	69.6+/−35.0	0.245
TMT B-A answers (n)	−0.5+/−1.1	−0.5+/−1.4	1.000
TMT B-A time (s)	57.7+/−46.8	43.9+/−32.5	0.336
OAT number trials needed (n)	29.5+/−14.7	23.5+/−12.1	0.222
OAT correct answers (n)	21.7+/−9.1	17.7+/−6.4	0.152
OAT errors (n)	7.7+/−6.7	5.8+/−6.1	0.425
Stroop test colour (n)	79.4+/−12.2	90.2+/−8.8	**0.002**
Stroop test reading (n)	97.9+/−4.7	100.6+/−11.9	0.417
Stroop test colour/reading (n)	52.5+/−19.3	67.6+/−13.5	**0.003**
Stroop interference (index)	9.5+/−15.7	20.6+/−12	**0.009**
Tower of London (n)	15.2+/−4.8	15.8+/−2.2	0.825
Tower of London total time (s)	448+/−170	330+/−76	**0.028**
Tower of London time first shot (s)	118+/−79	78+/−37	0.068

RCFT: Rey-Osterrieth Complex Figure Test.

TMT: Trail Making Test.

OAT: Object Alternation Task.

NA: Not applicable (either because no measure was available for healthy controls or because patients and controls were matched for these variables).

### Cerebral metabolic differences between OCD patients and HC

**Figure 1:**
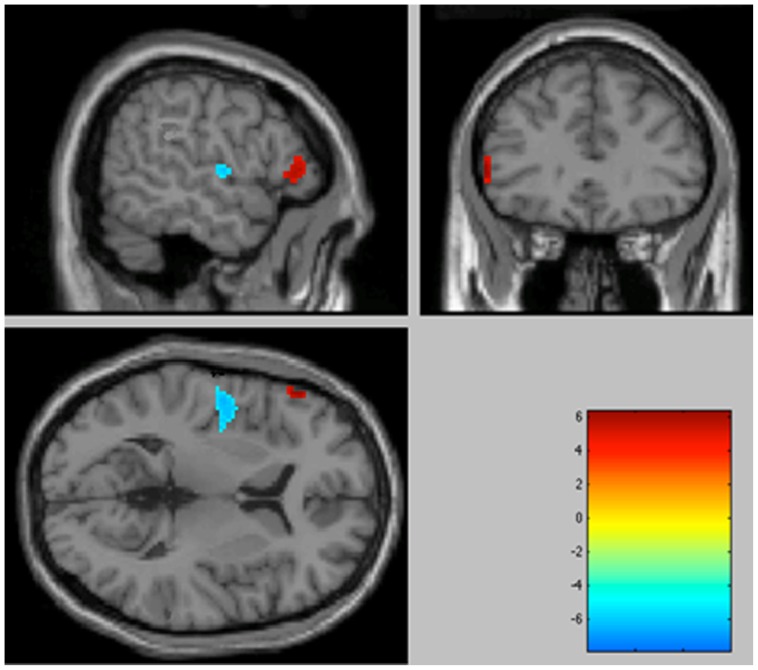
Significantly increased metabolic activity in the OCD patient group compared with healthy volunteers in the left inferior frontal gyrus (BA 45), associated with a decrease in activity in the left insula (BA 13) (*p*<0.001 uncorrected, colour bar represents *t* values). Sagittal, coronal and transversal views in projection onto brain slices of a standard MRI (x/y/z coordinates according to Talairach atlas).

The areas of significant differences found by comparing the OCD patients with the HC are shown in **Figures 1** **and** **2**. Coordinates are provided in **Table 2**.

**Figure 2:**
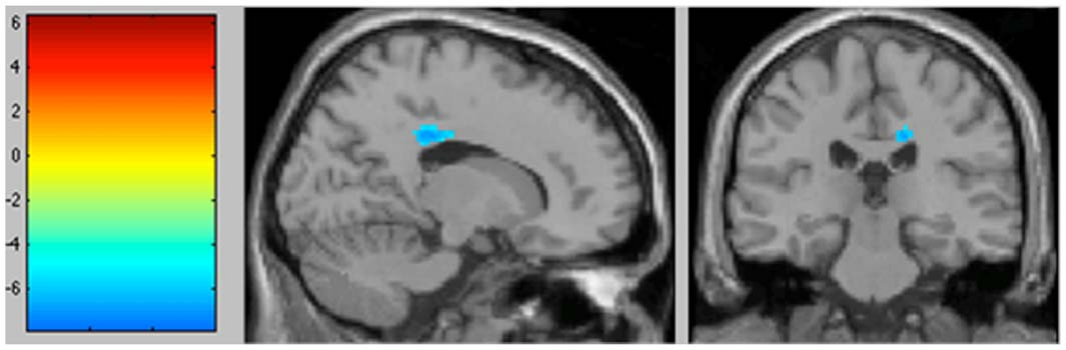
Significantly decreased metabolic activity in the OCD patient group compared with healthy volunteers in the right cingulate gyrus (BA 31) (*p*<0.001 uncorrected, colour bar represents *t* values). Sagittal and coronal views in projection onto brain slices of a standard MRI (x/y/z coordinates according to Talairach atlas).

**Table 2:** Comparison of regional glucose metabolism between OCD patients and healthy volunteers.

Region	Talairach coordinates x	Talairach coordinates y	Talairach coordinates z	*z* value	Voxel number
**Increases in OCD patients**					
Left inferior frontal gyrus, BA 45	−55	31	2	3.28	51
Right middle frontal gyrus, BA 9	40	19	29	3.19	53
**Decreases in OCD patients**					
Right cingulate gyrus, BA 31	16	−26	33	4	64
Right anterior cingulate gyrus, BA 24	12	−16	34	3.03	60
Left insula BA 13	−40	−6	−3	3.51	174

(*p*<0.001, uncorrected, *k*>50) (BA: Brodman area).

When we studied increases in metabolism in the OCD patients, two clusters were found to be significant at *p*<0.001 uncorrected. Hypermetabolism was observed in the left inferior frontal gyrus (Brodmann area (BA) 45) and the right middle frontal gyrus (BA 9).

When we studied decreases in metabolism in the OCD patients, three clusters were found to be significant at *p*<0.001, uncorrected. Hypometabolism was observed in the right anterior cingulate gyrus (ACG) (BA 31 and BA 24), and the left insula (BA 13).

Verbal (animal) fluency correlated positively with the left parietal lobe (inferior lobule (BA 40) and superior lobule (BA 7)) and right parietal lobe (precuneus (BA 7) and inferior lobule (BA 40)).

Verbal (animal) fluency correlated negatively with the left temporal lobe (middle gyrus (BA 39)) and left parietal lobe (angular gyrus (BA 7)).

The Stroop interference score correlated negatively with the right frontal lobe (superior gyrus (BA 11) and orbital gyrus (BA 47)), left parietal lobe (middle gyrus (BA 8)), and left frontal lobe (middle gyrus (BA 10) and superior gyrus (BA 11)). No positive correlations were observed between glucose metabolism and the Stroop interference score.

Tower of London completion times correlated positively with the left frontal lobe (inferior gyrus (BAs 45 and 47) and superior gyrus (BAs 8 and 9)), left parietal lobe (inferior lobule (BA 40) and angular gyrus (BA 39)), and right frontal lobe (inferior gyrus (BA 47)). No negative correlations were observed between glucose metabolism and Tower of London completion times.

These correlations are shown in **Tables 3** **,** **4** **,** **5** **and** **6**.

**Table 3:** Summary of the analysis of correlations between verbal fluency (animals) and glucose metabolism.

Region	Talairach coordinates x	Talairach coordinates y	Talairach coordinates z	*z* value	Voxel number
Left parietal lobe, inferior lobule, BA 40	−40	−36	40	4.68	592
Left parietal lobe, superior lobule, BA 7	−32	−58	64	4.55	592
Right parietal lobe, precuneus, BA 7	18	−60	52	3.84	123
Right parietal lobe, inferior lobule, BA 40	50	−32	46	3.66	92

(*p*<0.001, uncorrected, *k*>50) in 15 OCD patients. Increases in neuropsychological data are correlated with increases in glucose metabolism. (BA: Brodman area).

**Table 4:** Summary of the analysis of correlations between verbal fluency (animals) and glucose metabolism (*p*<0.001, uncorrected, *k*>50) in 15 OCD patients.

Region	Talairach coordinates x	Talairach coordinates y	Talairach coordinates z	*z* value	Voxel number
Left temporal lobe, middle gyrus, BA 39	−50	−68	28	4.00	279
Left parietal lobe, angular gyrus, BA 7	−46	−72	34	3.98	279

Increases in neuropsychological data are correlated with decreases in glucose metabolism. (BA: Brodman area).

**Table 5:** Summary of the analysis of correlations between Stroop interference and glucose metabolism (*p*<0.001, uncorrected, *k*>50) in 15 OCD patients.

Region	Talairach coordinates x	Talairach coordinates y	Talairach coordinates z	*z* value	Voxel number
Right frontal lobe, superior gyrus, BA 11	18	60	−18	5.05	510
Right frontal lobe, orbital gyrus, BA 47	14	24	−28	3.71	510
Left parietal lobe, middle gyrus, BA 8	−34	18	44	3.96	225
Left frontal lobe, middle gyrus, BA 10	−40	60	−4	3.71	122
Left frontal lobe, superior gyrus, BA 11	−46	50	−12	3.12	122

Increases in neuropsychological data are correlated with decreases in glucose metabolism. (BA: Brodman area).

**Table 6:** Summary of the analysis of correlations between Tower of London completion times and glucose metabolism (*p*<0.001, uncorrected, *k*>50) in 15 OCD patients.

Regions	Talairach coordinates x	Talairach coordinates y	Talairach coordinates z	*z* value	Voxel number
Left frontal lobe, inferior gyrus, BA 45	−48	26	20	5.52	296
Left frontal lobe, inferior gyrus, BA 47	−56	28	−6	3.40	296
Right frontal lobe, inferior gyrus, BA 47	56	26	−12	3.94	212
Left parietal lobe, inferior lobule, BA 40	−48	−56	36	3.86	212
Left parietal lobe, angular gyrus, BA 39	−42	−68	28	3.75	168
Left frontal lobe, superior gyrus, BA 8	−16	36	42	3.85	196
Left frontal lobe, superior gyrus, BA 9	−18	44	38	3.77	196

Increases in neuropsychological data are correlated with increases in glucose metabolism. (BA: Brodman area).

## Discussion

### Summary of evidence

In the OCD patients, executive functions were found to be slightly impaired, and modifications in glucose metabolism were observed in several frontal regions, namely the OFC (BA 45), the dorsolateral prefrontal cortex (DLPFC (BA 9)) and the ACG (BAs 24 and 31). These results support those of previous studies demonstrating that the PFC plays a central role in OCD [4], [5], [6], [7]. However, neuropsychological differences between the OCD patients and the HC were only subtle. Nor were they restricted to PFC resting-state metabolism. Thus, compared with HC, OCD patients were only behaviourally impaired on three tasks: verbal fluency, the Stroop test and the Tower of London. Furthermore, these impaired executive functions were correlated with glucose modifications not just in the PFC but also in the parietal and temporal lobes. This indicates that, contrary to the hypothesis suggested by STN DBS efficacy, OCD is not an entirely prefrontal pathology. Other regions are also involved and may help to explain the complexity of the OCD syndrome, as suggested by Menzies et al. [29] The differences we observed in resting-state cerebral metabolism between the OCD patients and HC in the insula and the dorsoparietal cortex certainly argue in favour of this theory and suggest that OCD networks should be revisited.

### OCD networks revisited

One of the first studies to use ^18^FDG-PET to assess OCD patients observed increased metabolism in the left OFC in patients compared with normal participants [4]. Several studies have yielded similar findings, reporting increased functional activity in the orbitofrontal area in resting-state ^18^FDG-PET studies, either bilaterally [4], [5] or restricted to the left [6] or right side [7]. Other studies, however, have failed to find any hypermetabolism in the OFC [30]. The OFC is a large region that encompasses both rostral (BAs 10 and 47) and medial (BAs 11, 12, 13 and 14) areas. It plays an important role in emotion and social behaviour, and has been described as allowing for the highest level of integration for emotional information processing [31], [32]. When this area is functionally hyperactive, the natural process of weighing up the consequences of immediate action may become overactive, leading to uncontrolled thoughts and behaviour [3].

The right DLPFC (BA 9) was hypermetabolic in our study. The DLPFC is associated with the high-level, executive processes needed for voluntary, goal-directed behaviour. It is also involved in cognitive control, namely the ability to voluntarily focus awareness on certain sensory inputs, thoughts or actions, and to refocus awareness on other inputs according to changes in the environment [33]. In a spectroscopy study, Russell et al. [34] found that levels of N-acetyl-aspartate, a marker of neuronal integrity, were significantly increased in the DLPFC in 15 treatment-naïve cases of OCD. This region is known to be involved in OCD [3], [29]. We suggest that DLPFC hyperactivity could reflect both the lack of cognitive control over obsessive thoughts (contamination or doubt obsessions) and its consequences on attentional resources. Indeed, cognitive control refers to high-level executive processes that allocate cognitive resources to relevant goals and divert cognitive resources away from irrelevant goals. Impaired executive functioning can be a consequence of the hyper-allocation of cognitive resources to obsessive thoughts. Thus, the deficit in executive functions and the hypermetabolism of the DLPFC would reflect the misuse of cognitive resources [35], [36] in OCD.

We found decreased glucose metabolism in the right side of the ACC (BAs 24 and 31), in contrast to previous results reported by Swedo et al. [6] and Perani et al. [37] Animal and neuropsychological studies have indicated that this region is involved in cognitive processes such as attention, motivation, working memory, reward and error detection, problem-solving and action-planning [3], [38]. Bush et al. [38] have suggested that it may also play a role in the management of cognitive and emotional information. A dysfunction of this area could explain several clinical symptoms presented by OCD patients. The cognitive difficulties of OCD patients could thus be related to a lack of motivation corresponding to ACC hypoactivity.

We observed correlations between the metabolism of the parietal lobe (Bas 40 and 7) and impaired executive functions. The parietal lobe is thought to be involved in functions such as attention and spatial perception, as well as response inhibition [39]. Nordahl et al. found [5] a basal hypometabolism of this structure in OCD patients. This may account for the clinical symptoms observed in OCD patients, who display deficits such as a focusing on particular ideas.

Our data are in line with those of van den Heuvel et al., who suggested that the OFC is not the sole brain area implicated in OCD [40]. They found that some OCD symptom dimensions were related to different parts of the brain. They also reported that the symmetry/ordering scores of OCD patients were negatively correlated with the grey matter volume of the bilateral parietal cortex and the right motor and left insular cortices.

We also found a modification in glucose metabolism in the insula, with hypometabolism in the OCD patients compared with the HC. The insular cortex is involved in empathy, compassion and interpersonal characteristics such as fairness and cooperation. As such, it plays an important role in social emotions, defined here as affective states that arise when we interact with other people, and which depend on social context [41]. Paulus and Stein have suggested that the insula computes an “interoceptive prediction error”, signalling a mismatch between actual and anticipated bodily arousal, and evoking subjective anxiety and avoidance behaviour [42]. These studies clearly show that the insular cortex is important for linking emotions to cognitive processes and behavioural responses. On the whole, in line with Menzies at al. [29], our results suggest that OCD stems from a dysfunction not only of the OFC but also of a larger region including the parietal lobe and the DLPFC. Moreover, the insula is probably part of this network and therefore merits closer inspection.

### Increased glucose activity in the PFC: a potential therapeutic marker for OCD?

All these metabolic results are concordant with the anatomical-functional approach to OCD, linking cortical structures with subcortical ones. We found impaired glucose metabolism in three cortical regions involved in the three basal cortico-subcortical circuits that were first defined by Alexander et al. [43]. In particular, our replication of a previous finding of increased glucose metabolism in PFC, which seems to be attenuated by treatments such as DBS [1], [2], [44] and, potentially, repetitive transcranial magnetic stimulation (rTMS) targeting the OFC (Millet et al. in preparation), leads us to regard the PFC as a potential functional neuroimaging marker of this disorder. Additional randomized controlled trials using a PET scan approach are needed to confirm this hypothesis.

### Limitations

Our exploratory study, like all hypothesis-generating research, ran the risk of false-positive or false-negative findings, as indicated by Ioannidis [45]. This is a risk run by each and every neuroimaging study of OCD. Moreover, it is not easy to set our study results against previous research findings. As Whiteside et al. underlined in their meta-analysis [46], the OCD groups investigated in previous studies are far from homogeneous, in terms of handedness, IQ, demographic characteristics [29], comorbidity (particularly depression or anxiety), OCD subtypes, disease duration and current treatment. Nevertheless, most of our results do actually replicate previous findings. This could be regarded as a form of external validity, given that our study was based on sound methodology. None of the patients included in our sample had psychiatric comorbidities. Furthermore, they were matched with HC for age, sex and education level, and all participants underwent the ^18^FDG-PET scan in the same standardized conditions.

We cannot completely rule out treatment exposure as a potential confounding factor, possibly affecting resting-state brain activity and cognitive function. Even so, we limited this bias as far as possible, as patients had to have been without serotonin reuptake inhibitors for at least one month and without cognitive-behavioural therapy for the past year. One other possible confounding factor was the presence of depressive syndrome, suggested by the higher MADRS scores in the OCD group, as depressed patients exhibit impaired cognitive functions [47]. We did, however, limit this bias from a qualitative point of view, by excluding patients with major depressive disorder. Moreover, from a clinical point of view, OCD patients score higher on the MADRS because the scale contains items that are not specific to depression and pertain to anxiety symptoms as well [48]. It is therefore difficult to distinguish between OCD symptomatology and depressive symptomatology. Thus, from a quantitative point of view, we used a cut-off score to limit the impact of this possible confounding factor.

An ideal study would contrast controls with OCD patients 1/in a completely unmedicated state, but this is not realistic from an ethical point of view, and 2/free from depressive symptoms, as assessed by depression rating scales, but this is unrealistic from a clinical point of view.

Finally, we unfortunately failed to assess age at onset, even though it would have been interesting to compare early-onset versus late-onset patients. Preliminary findings suggest that late, but not early onset of OCD is associated with abnormally low central serotonin transporter availability [49], which could have an impact on brain metabolism at rest.

## Conclusion

Our study revealed mildly impaired executive functions and abnormal resting-state glucose metabolism. Results confirmed the involvement of the PFC, especially the OFC, suggesting that it should be regarded as a neurofunctional marker of the disorder and a potential therapeutic target. The slight cognitive impairment highlighted by the neuropsychological tests suggests that cognitive dysfunction should be regarded more as a consequence of obsessive thoughts than a cause.

In order to deepen our understanding of OCD, the two obvious next steps are 1/analyzing these characteristics from a dynamic perspective in order to explore the network involved in OCD, and 2/combining structural (diffusion tensor imaging) and functional neuroimaging techniques in order to distinguish the primary dysfunctional areas from those involved in the adjustment of brain functions to the disorder.
